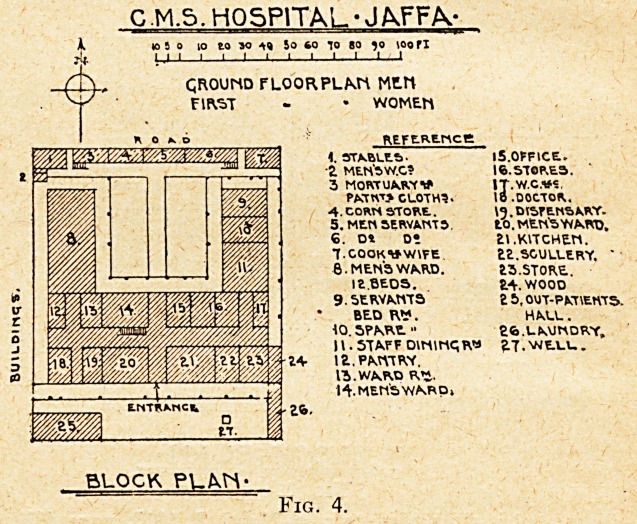# Some Foreign Hospitals in Jerusalem

**Published:** 1918-06-22

**Authors:** 


### HOSPITALS IN THE HOLY LAND.

### SOME FOREIGN HOSPITALS IN JERUSALEM.

THE commission to design and build a hospital at Jerusalem for the Church of England Missions to the Jews (which completed hospital was fully described and

FIG. 1.

illustrated in THE HOSPITAL of March 2, 1918, page 461) led to a prolonged visit in order to study the local conditions both of organisation and use and also of the building materials and methods that were available. There are several considerable establishments belonging to European national communities and Churches upon the modern buildings of which large sums have been expended and with some architectural pretension. Their purpose was the historical one of providing for pilgrims, and their existence indicates the dangers to health and liability to accidents by which pilgrimage is still accompanied.

### THE FIRST MEDICAL MISSION.

Within the city walls, through streets inaccessible both to carriages and horseshoes, amidst most insanitary and impossible surroundings, the English Church Missions to the Jews had established, about seventy years ago, what was probably the first medical mission of modern times. On the upper part of Mount Zion, in adapted native buildings, long rooms, low vaulted and dimly lighted, opened out of the upper storeys of low courtyards upon the flat stone roofs of the adjacent buildings; the cavern-like dispensary and densely crowded outpatients' department were below, and in the narrow alleys around the patients accumulated during the night for the early visit of the doctor. His consulting-room seemed to resemble the guest or common-room of a caravanserai; here the patients were sorted out in the corners and according to their languages by the dragoman, conducted to the nurses for polyglot entries on cards, and passed on to the doctor, sitting near the window and partially screened, for the examination and for the charm or prescription.

This old centre of mercy within the city walls achieved and maintained a reputation, through the devotion and skill of its staff, over an area in Syria, Arabia, and the near Asian East that the languages and costumes of the patients remarkably illustrated.

### NEARLY A MILE OF HOSPITALS.

Outside the city, beginning from the Jaffa gate, the series of national hospitals begins, extending for nearly a mile along the road that skirts the modern suburbs and squalid "colonies." The Greek hospital is a large quadrangular block with an internal court; the office and dispensing-rooms are in front on either side of the entrance, the nurses' rooms and kitchens at the back, and the wards on the sides; looking into the court on the front side are the consulting and operating chambers, and opposite the latrines on either side of a spacious stair to the flat roofs. All the windows of the rooms on each side of the quadrangle look into the dusty streets; the wards are small, about five beds in each, three opposite to and two under the windows, and all the rooms, wards, kitchens, stores, latrines (which were foul), dispensary, and offices open into the enclosed passage around the central court.

FIG. 2—VIEW OF FRENCH HOSPITAL, JERUSALEM (ON LEFT). ON THE RIGHT IS THE NUNNERY.

**This Photograph was taken in 1898, and shows the Kaiser entering Jerusalem on a white charger.**

Though the building was lofty and only of one storey the vaulted roofs made the wards low internally. The

**HOSPITALS IN THE HOLY LAND**—(*continued*).

one redeeming feature was the use made of the extensive stone flats above for airing the patients by means of the capacious stair. The simple architectural scheme of the quadrangle and the regular disposition of apartments, irrespective of their several purposes, the close stuffiness of the continuous corridors and the external aspect on the dust-laden roads provided elementary lessons in what to avoid, and a striking absence of hospital science in planning.

ROOMS VENTILATING INTO THE CENTRAL HALL.

The Russian hospital (see Fig. 1) is a detached building within the large and dignified compound of the great hospice and has an equally simple plan. A long, vaulted central hall, lighted only by closed windows at the ends, divides the women's rooms from those of the men; to term wards the three square rooms devoted on each side to patients might convey a wrong impression; these are separated from each other by narrow rooms for single beds or service, the "wards" each containing five beds; the latrines are within the block, next to the roof staircase at the end, and a lofty wooden screen from end to end divides the hall used as a day-room by the men and women. Into this central hall all the rooms open and ventilate, and it will not be a matter of surprise that occasionally the building has had to be closed for disinfection. The idea seems to have been that the colder-blooded pilgrims from the Steppes needed enclosure and mutual warmth.

A subsequently attached isolation block consisting of five small rooms, three of which open on to a common passage, has been erected to supplement this hospital.

The floors throughout were of wood painted and polished, the ceilings vaulted, and the wide staircase was of cast iron.

WARDS, CONFINED AND STUFFY VAULTS.

The French hospital of St. Louis is of a different architectural composition. It consists of a long central block

FIG. 3.

with transverse wings at each end, four square wards for women, with windows on one side, form the main building, and two oblong wards with windows at one end, for men, occupy one wing; the other wing is given up to a large chapel. The dispensary and consulting-room adjoin the former wing, and out-patients wait outside; an isolation block is provided. This is a large and handsome erection, more recent than the Greek or Russian hospitals, but with the same faults of academic prejudice in design, accepting with the arched stone ceilings the limitation of width between walls and loss of clear height that make the wards into confined and stuffy vaults.

A STRANGELY UNSUITABLE PLAN.

The German hospital (see Fig. 3) of the Kaiserswerth Deaconesses is more modern still. It is situated at the corner of two roads and is entered at the angle. A central internal corridor, L-shaped, impinges on the entrance and is lighted at the far ends; opposite the entrance is an octagon hall occupying the internal angle; on either side of the corridors are the wards, holding three

FIG. 4.

beds each, oblong, with windows on the outer long side, but all opening into the practically unventilated internal passages. There are two main floors with a basement in which are the out-patients' rooms and kitchens. The latrines are at the ends of the corridors, and, like all the doors, are under observation from the central entrance. The operating-room is over the entrance and the chapel opposite its door over the hall. The roofs are flat, and a laundry and drying-shed for winter use are on that storey. This building is constructed with iron girders arched between in stone, and the waste of arches and abutments is thus avoided. The windows next to the roads on the outside are exposed to the dust nuisance, and those on the rear, on the internal angle, are deprived of free air circulation. The plan is again an original architectural scheme laid down on academic lines, without stint of money, but strangely unsuitable for hospital purposes either in the East or anywhere else.

RAIN-WATER STORED IN BASEMENT.

The Rothschild hospital is more typical of its purpose, though presenting serious and obvious defects. It consists of a central block for administrative purposes, with attached wards at either end of the internal unventilated corridor that connects them, and in which the latrines are situated. The wards are cross-ventilated; 35 feet long by $17\frac{1}{2}$ feet broad, and 17 feet in height. The out-patients and dispensary are in the basement, and a good disinfecting plant is provided. An important feature of the building is that the rain-water store cisterns form the basements of the wards, an economical scheme from the point of view of the planner, but the effect on the temperature or the atmospheric condition of the building of this arrangement has been considered unwholesome. Whether the non-success of the building as a hospital

(*Concluded on page* 250.)

### HOSPITALS IN THE HOLY LAND.— (*Concluded from page* 252).

is due to this cause alone may be doubted in view of the corridor plan; but the cross-current provided in the wards has not availed to make them successful. This hospital, like the others, is wholly without verandah protection, in this case specially demanded for the ward windows.

### Homeliness Without Design.

The Moravian Leper House, isolated and distant, is a building of pathetic purpose and of a simple Eastern homeliness, without design either in the academic or hospital sense. It seems to defy any sanitary analysis, but does its work of mercy nevertheless as an asylum for its pitiably incurable inmates. The inmates, however, include the entirely healthy family of the house-parents, who have reared their own children within its walls, guarded only by personal cleanliness from the danger of the patients. The site is high, open, and free, necessarily, from the dust of the public roads. The buildings surround a large open court that contains the cistern, and form a rough quadrangle. On the left of the central entrance are the house-parents' rooms, on each of the two floors and on the right the wards are small apartments connected by wide arches and forming a series of bright and well-ventilated rooms. The men and women are placed on opposite sides of the court, with properly detached latrine blocks. Between the wards a large hall or day-room occupies one side, with the chapel above, and on the opposite side are the kitchen and offices, with a supplementary court. Fresh air, freedom of circulation, an open iron gate being the only separation between the family quarters and the patients, and a pleasant uninstitutional homeliness, after the Eastern pattern, mark the whole of the arrangements and are characteristic of the cheerful family life maintained in the presence of deplorable disfigurement and suffering. In its local manner it approaches our more recent appreciation of village or colony treatment for chronics and incurables of other kinds at home, and from the Christian devotion of its managers it derives its sanitary as well as other success.

### Some Mission Hospitals in Palestine.

Hospitals have been erected at many stations by various English and Scottish missionary bodies; these each have special interest and merit long description and much advertisement. Jaffa has a commodious, economical, and practicable English hospital (see Fig. 4), a practical adaptation of a cottage hospital, built with verandahs and airy wards. Hebron has a Scotch mission and modern hospital, while at Gaza is a well-found hospital of the Church Missionary Society (see The Hospital, Dec. 15, 1917, p. 222). But these are only a few of the many scattered throughout Palestine, all of which we hope will soon again be in full activity.

## Figures and Tables

**Fig. 1. f1:**
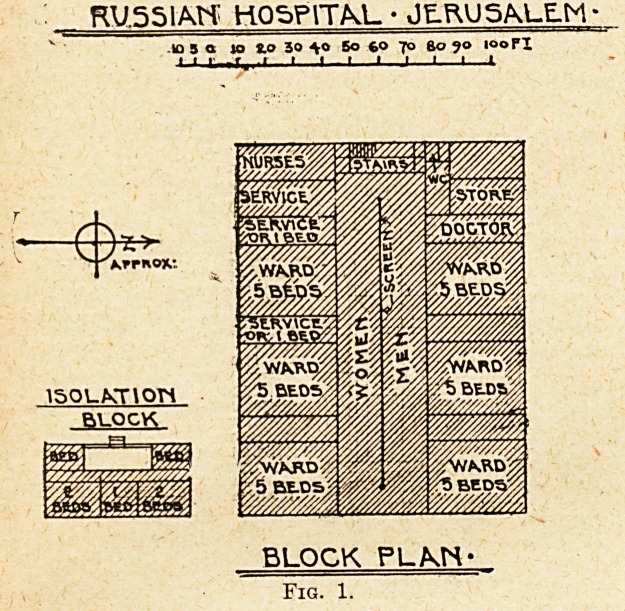


**Fig. 2. f2:**
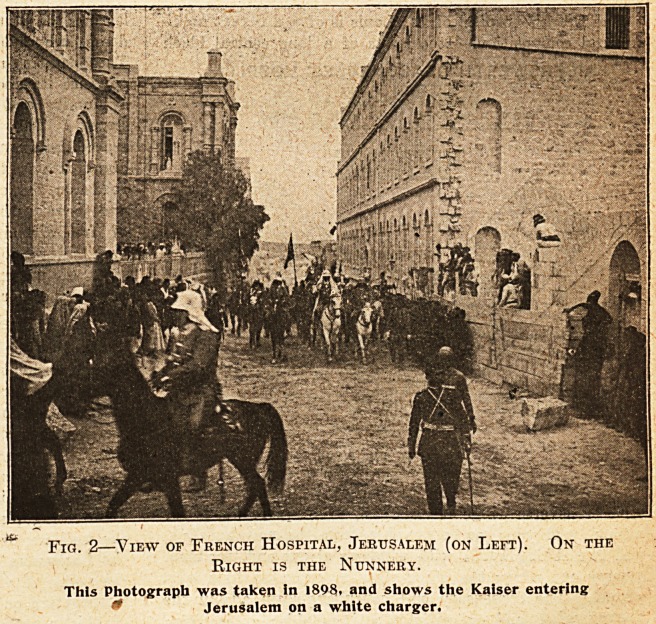


**Fig. 3. f3:**
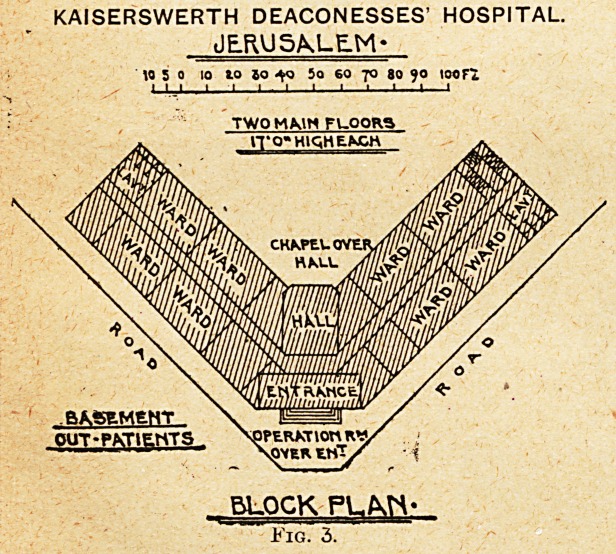


**Fig. 4. f4:**